# Transcatheter Edge-to-Edge Repair for Mitral Valve Systolic Anterior Motion in Complex Left Ventricular Outflow Tract Phenotypes: A Case Series

**DOI:** 10.1016/j.jscai.2026.105622

**Published:** 2026-09-10

**Authors:** Mark J. Zorman, Smitha Narayana Gowda, Ratnasari Padang, Jeremy J. Thaden, Mayra Guerrero, Mackram F. Eleid

**Affiliations:** Department of Cardiovascular Medicine, Mayo Clinic, Rochester, Minnesota

**Keywords:** case report, hypertrophic cardiomyopathy, left ventricular outflow tract obstruction, systolic anterior motion, transcatheter edge-to-edge repair

## Abstract

Dynamic left ventricular outflow tract obstruction due to systolic anterior motion of the mitral valve can arise across diverse anatomic substrates. We report 5 consecutive patients with systolic anterior motion–mediated left ventricular outflow tract obstruction treated with the PASCAL Ace (Edwards Lifesciences) device at a single center. Technical success, mitral regurgitation reduction to ≤2+, relief of left ventricular outflow tract obstruction, and symptomatic improvement to New York Heart Association class I to II were achieved in all patients, without clinically significant mitral stenosis.

## Introduction

Dynamic left ventricular outflow tract (LVOT) obstruction arises from abnormal interaction between the interventricular septum, mitral valve, and left ventricular geometry.^1^^,^^2^ It most commonly manifests as systolic anterior motion (SAM) of the anterior mitral leaflet, driven by drag forces of the systolic outflow jet, which disrupts leaflet coaptation and results in posteriorly directed mitral regurgitation (MR).^1^, ^2^, ^3^ Although hypertrophic cardiomyopathy (HCM) is the archetypal substrate, SAM-mediated obstruction also arises in other settings of aberrant mitral-septal interaction.^1^^,^^2^^,^^4^

Surgical myectomy remains the gold standard for patients with symptomatic LVOT obstruction refractory to medical therapy.^1^^,^^5^ Septal ablation and septal scoring along the midline endocardium offer percutaneous alternatives for patients at high surgical risk,^6^ although neither approach directly addresses the mitral apparatus and SAM-related MR. Mitral transcatheter edge-to-edge repair (M-TEER) has emerged as a strategy simultaneously targeting SAM-related MR and LVOT obstruction, but clinical experience remains limited.^7^

We report 5 patients with symptomatic SAM-mediated dynamic LVOT obstruction across heterogeneous anatomic substrates treated with M-TEER at Mayo Clinic Rochester between November 2022 and December 2025. Baseline, procedural, and follow-up characteristics are summarized in Table 1, with imaging in Figures 1 to 7 and Supplemental Videos 1 to 5. Two illustrative cases are described below.

**Table 1:** Baseline, procedural, and follow-up characteristics of patients undergoing M-TEER for SAM-mediated dynamic LVOT obstruction

Case	Age/sex	Substrate	Prior interventions	Preprocedural physiology	M-TEER strategy	Procedural result	Follow-up
1	71 M	HCM; elongated anterior mitral leaflet	Alcohol septal ablation; extended septal myectomy	Mild resting findings but severe provocable LVOT obstruction (>150 mm Hg) and severe SAM-mediated MR (Figure 1A-D)	1 PASCAL Ace, mid A2-lateral P2	SAM resolved; mild MR; no significant LVOT gradient; MVG 5 mm Hg (Figure 2A-E; Supplemental Video 1)	NYHA I-II at 1 year; mild MR; no recurrent obstruction
2	82 F	HCM; post-TAVR SAM and LVOT obstruction	2 prior alcohol septal ablations; pacemaker; TAVR	SAM-mediated MR with P2 prolapse following TAVR (Figure 3A-B). Resting LVOT gradient 38 mm Hg, increasing to 52 mm Hg with Valsalva; severe SAM-mediated MR with provocation (Figure 4A-B)	1 PASCAL Ace, A2-P2	MR trivial; improved LVOT obstruction; MVG 4 mm Hg (Figure 3C-D; Supplemental Video 2)	NYHA II and no changes from procedural TTE at 1 y
3	72 M	Basal septal hypertrophy; P2 prolapse	None; high surgical risk due to severe COPD	Severe MR; resting LVOT gradient 15 mm Hg; septum 16 mm (Figure 5A)	1 PASCAL Ace, lateral A2-P2	MR severe to trivial; LVOT gradient resolved; MVG 5 mm Hg; MVA 6.0 to 3.2 cm^2^ (Figure 5B-D; Supplemental Video 3)	Trivial MR and no LVOT gradient at 1 y
4	72 M	SAM after surgical mitral valve repair	Robotic mitral valve repair with posterior annuloplasty band	Severe SAM-related MR medial to A2-P2; LVOT gradient 49 mm Hg, 80 mm Hg after ectopy (Figure 6A)	1 PASCAL Ace, A3-P3	SAM eliminated; mild MR; MVG 5 mm Hg (Figure 6B-D; Supplemental Video 4)	NYHA I at 3 months; postprocedural TTE unchanged at 30 d
5	81 M	HCM; post-TAVR SAM and LVOT obstruction	Alcohol septal ablation; ICD; TAVR	Post-TAVR resting LVOT gradient 85 mm Hg; moderate-severe SAM-mediated MR; worsened with nitroglycerin (Figure 7A, D)	1 PASCAL Ace, A2-P2	Marked SAM reduction; trivial MR at rest and with nitroglycerin; MVG 3 mm Hg (Figure 7B-C, E; Supplemental Video 5)	NYHA II at 30 d

LVOT gradients are maximum instantaneous Doppler gradients unless otherwise specified.

COPD, chronic obstructive pulmonary disease; HCM, hypertrophic cardiomyopathy; ICD, implantable cardioverter-defibrillator; LVOT, left ventricular outflow tract; M-TEER, mitral transcatheter edge-to-edge repair; MR, mitral regurgitation; MVA, mitral valve area; MVG, mean mitral valve gradient; NYHA, New York Heart Association; SAM, systolic anterior motion; TAVR, transcatheter aortic valve replacement; TTE, transthoracic echocardiography.

**Figure 1:**
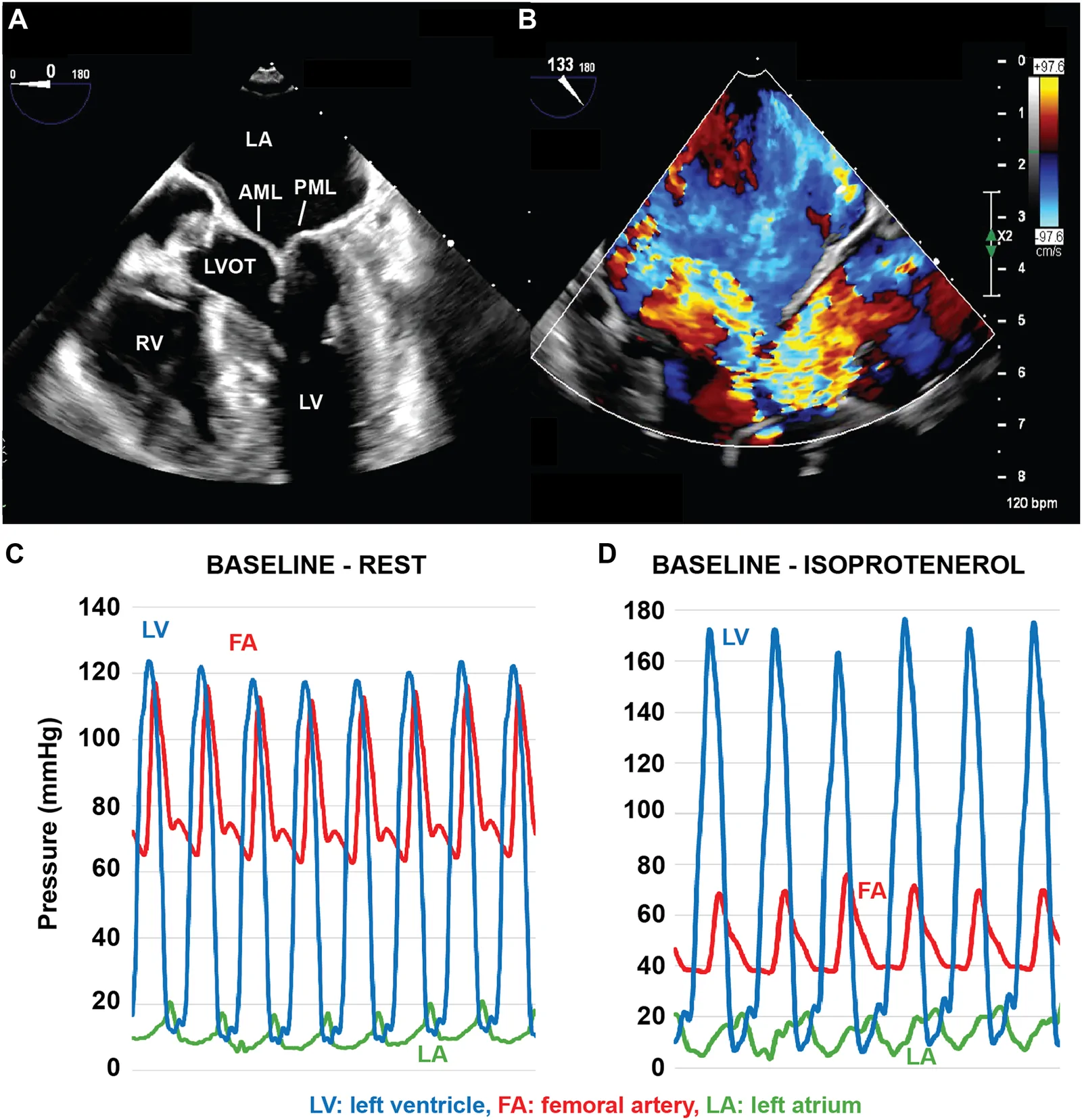
**Systolic anterior motion (SAM) of the anterior mitral leaflet resulting in LVOT obstruction and posteriorly directed MR (case 1).** (**A**) TEE midesophageal long-axis view in late systole showing SAM of an elongated AML. (**B**) Isoproterenol provocation showing dynamic LVOT obstruction and severe posteriorly directed MR. (**C**) Invasive hemodynamic assessment at rest showing mild LVOT obstruction and near-normal left atrial V wave with mild sedation. (**D**) Invasive hemodynamic assessment with isoproterenol provocation shows around 100 mm Hg LV-FA gradient, elevated LA V waves and systemic hypotension, due to dynamic LVOT obstruction and severe SAM-mediated MR. AML, anterior mitral leaflet; FA, femoral artery; LA, left atrium; LV, left ventricle; LVOT, left ventricular outflow tract; MR, mitral regurgitation; PML, posterior mitral leaflet; RV, right ventricle; SAM, systolic anterior motion; TEE, transesophageal echocardiography.

**Figure 2:**
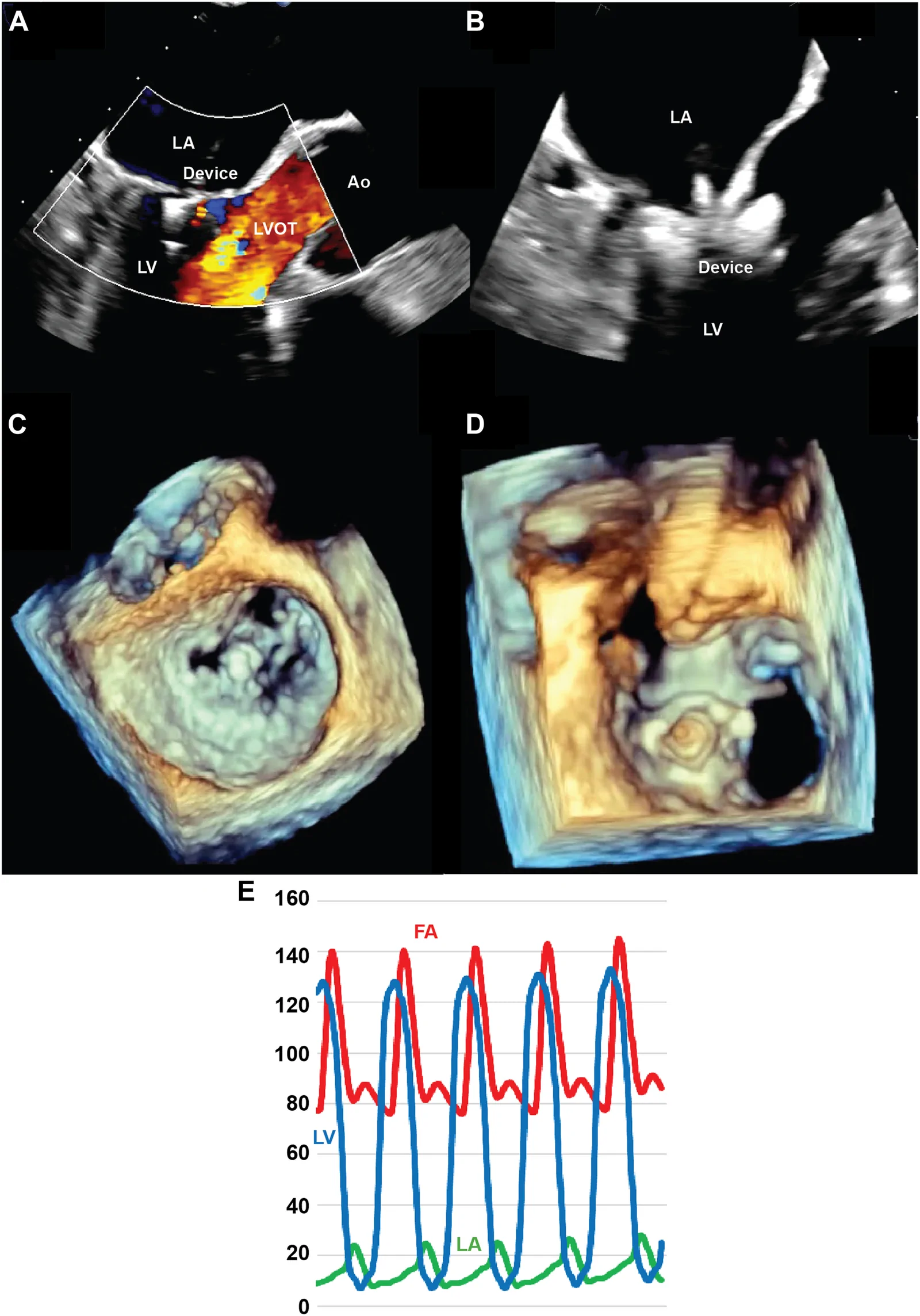
**Mitral TEER with PASCAL Ace device and resolution of dynamic LVOT obstruction (case 1).** (**A-B**) PASCAL Ace device positioned between mid A2 and lateral P2 scallops with resolution of SAM, mild residual MR, and no significant LVOT obstruction. Three-dimensional TEE en-face view of the mitral valve after PASCAL Ace device deployment in systole from the LA aspect (**C**) and diastole from the ventricular aspect (**D**). (**E**) Invasive hemodynamic assessment with isoproterenol provocation following device deployment confirmed resolution of significant LV-FA gradient and a reduction of LA pressure indicative of resolved LVOT obstruction. Ao, aorta; FA, femoral artery; LA, left atrium; LV, left ventricle; LVOT, left ventricular outflow tract; MR, mitral regurgitation; SAM, systolic anterior motion; TEE, transesophageal echocardiography.

**Figure 3:**
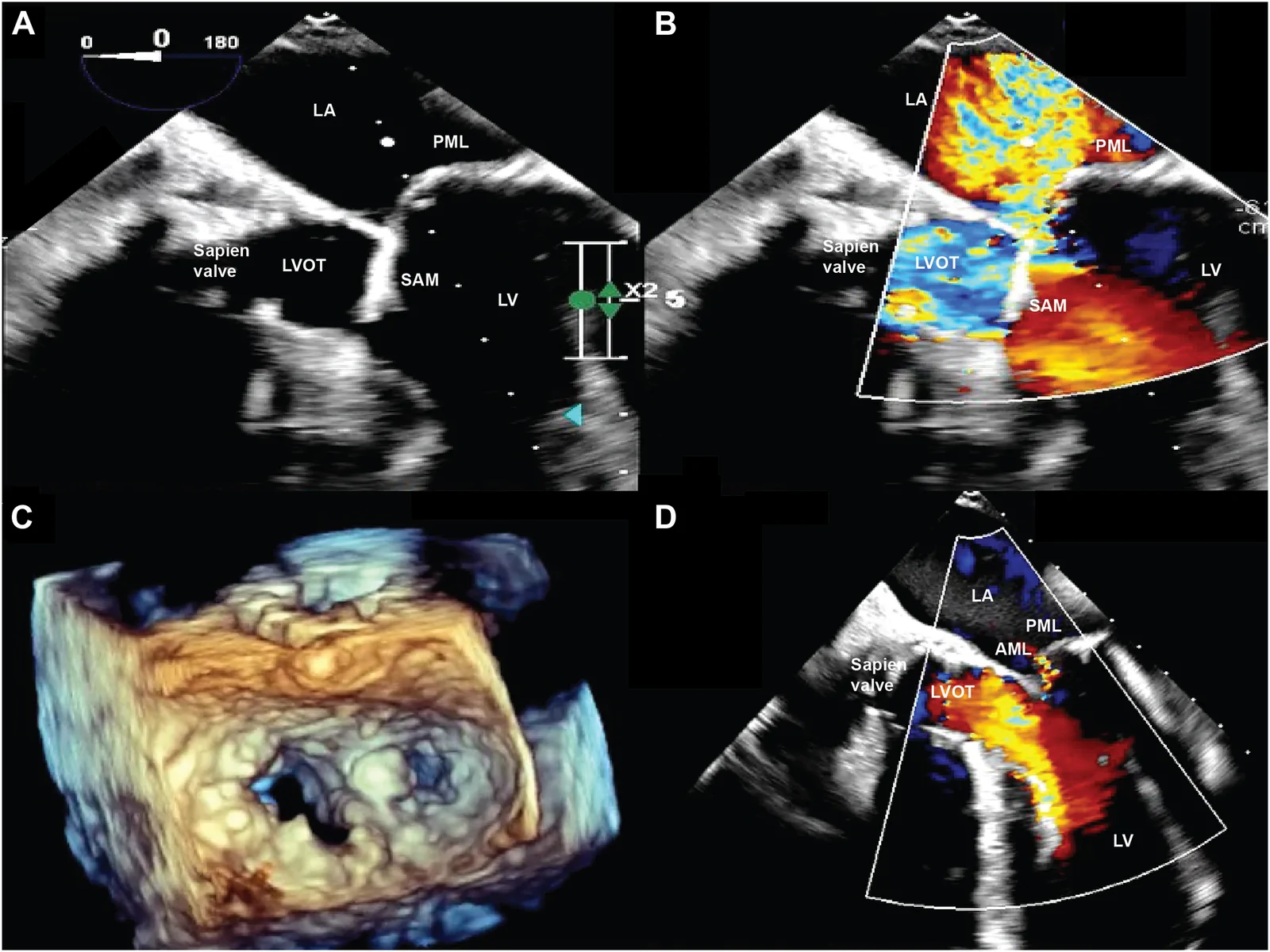
**SAM-mediated LVOT obstruction following TAVR treated with mitral TEER (case 2).** (**A**) Midesophageal long-axis TEE view demonstrating SAM of the mitral leaflets toward the interventricular septum, narrowing the LVOT in the presence of a 23-mm Sapien 3 Ultra aortic bioprosthesis. (**B**) Color Doppler imaging showing flow acceleration and turbulence within the LVOT caused by SAM, associated with severe MR. (**C**) Three-dimensional TEE en-face view of the mitral valve after PASCAL Ace deployment in diastole. (**D**) Color Doppler showing resolution of LVOT obstruction with laminar LVOT flow following mitral TEER and trivial MR.; AML, anterior mitral leaflet; LA, left atrium; LV, left ventricle; LVOT, left ventricular outflow tract; PML, posterior mitral leaflet; SAM, systolic anterior motion of mitral leaflets.

**Figure 4:**
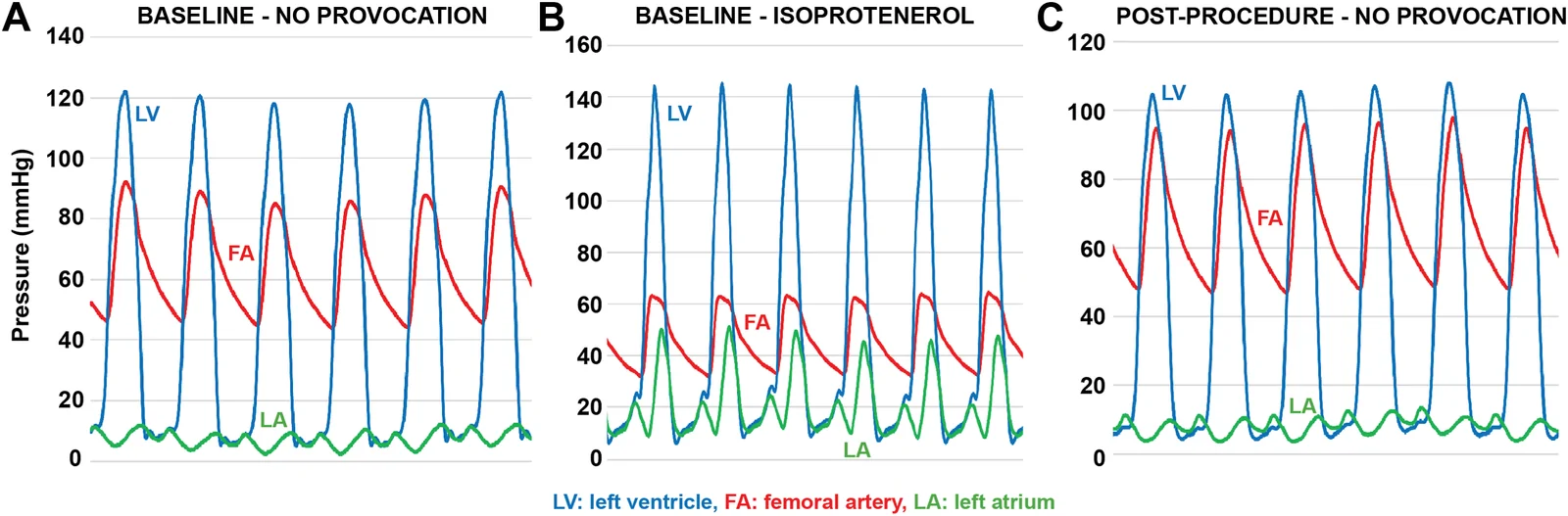
**Invasive hemodynamics demonstrating SAM-mediated LVOT obstruction before and after mitral TEER (case 2).** (**A**) Simultaneous baseline traces demonstrating a systolic LV-FA pressure gradient with normal LA pressure. (**B**) Isoproterenol provocation augmented the LVOT gradient and elevated LA V wave, suggesting severe SAM-mediated MR. (**C**) Following mitral TEER, the LV-FA gradient decreased with reduction in LA pressure, consistent with relief of SAM-mediated LVOT obstruction and resolution of SAM-mediated MR. FA, femoral artery; LA, left atrium; LV, left ventricle.

**Figure 5:**
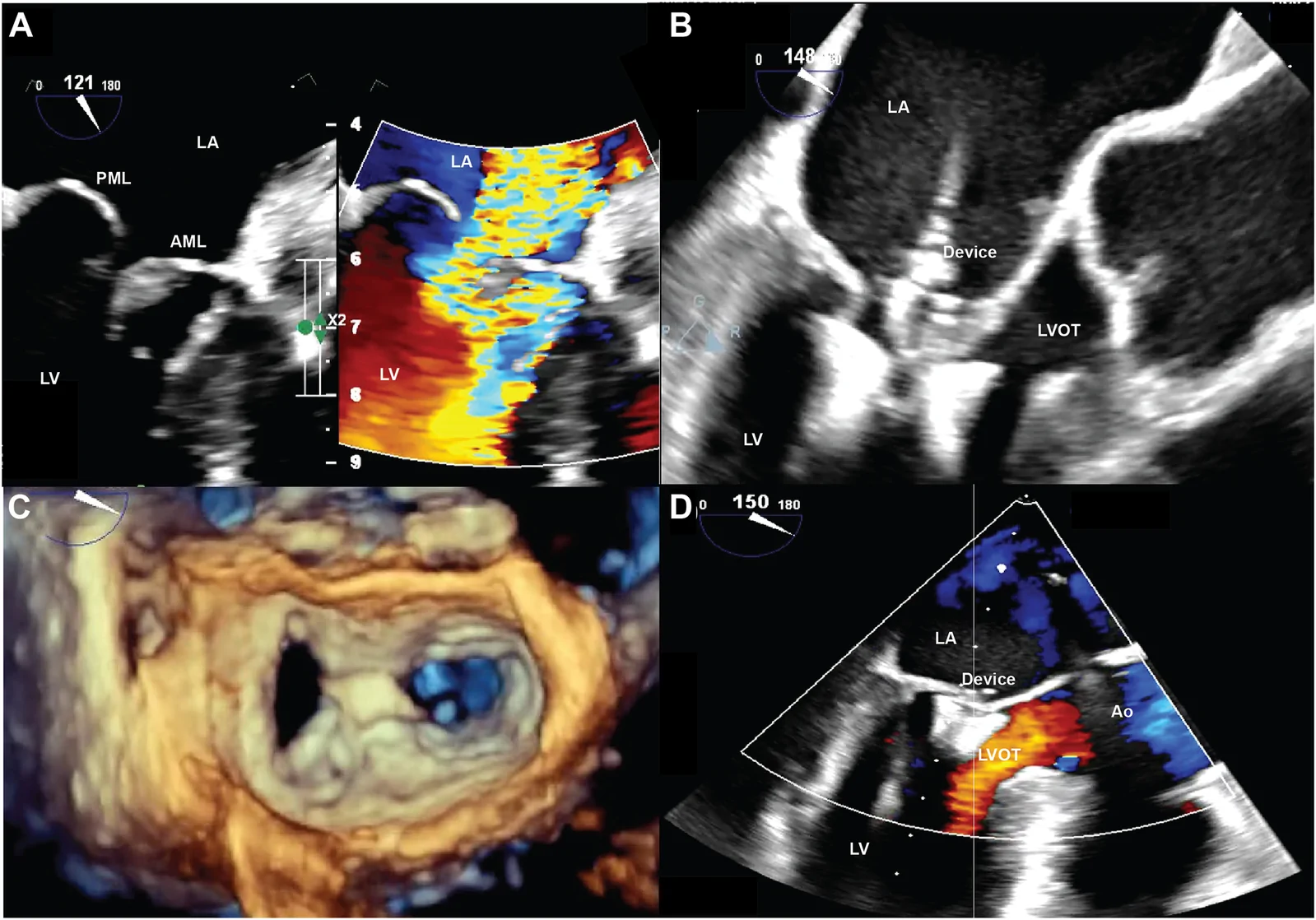
**Mitral TEER in a patient with basal septal hypertrophy and SAM (case 3).** (**A**) TEE demonstrating severe MR in the setting of posterior (P2) mitral leaflet prolapse, basal septal hypertrophy, and SAM. (**B**) PASCAL Ace device positioning between A2 and lateral P2 scallops. (**C**) Three-dimensional TEE en-face view of mitral valve after PASCAL Ace device deployment in diastole. (**D**) No residual MR and laminar LVOT flow after device deployment. Ao, aorta; AML, anterior mitral leaflet; LA, left atrium; LV, left ventricle; LVOT, left ventricular outflow tract; PML, posterior mitral leaflet.

**Figure 6:**
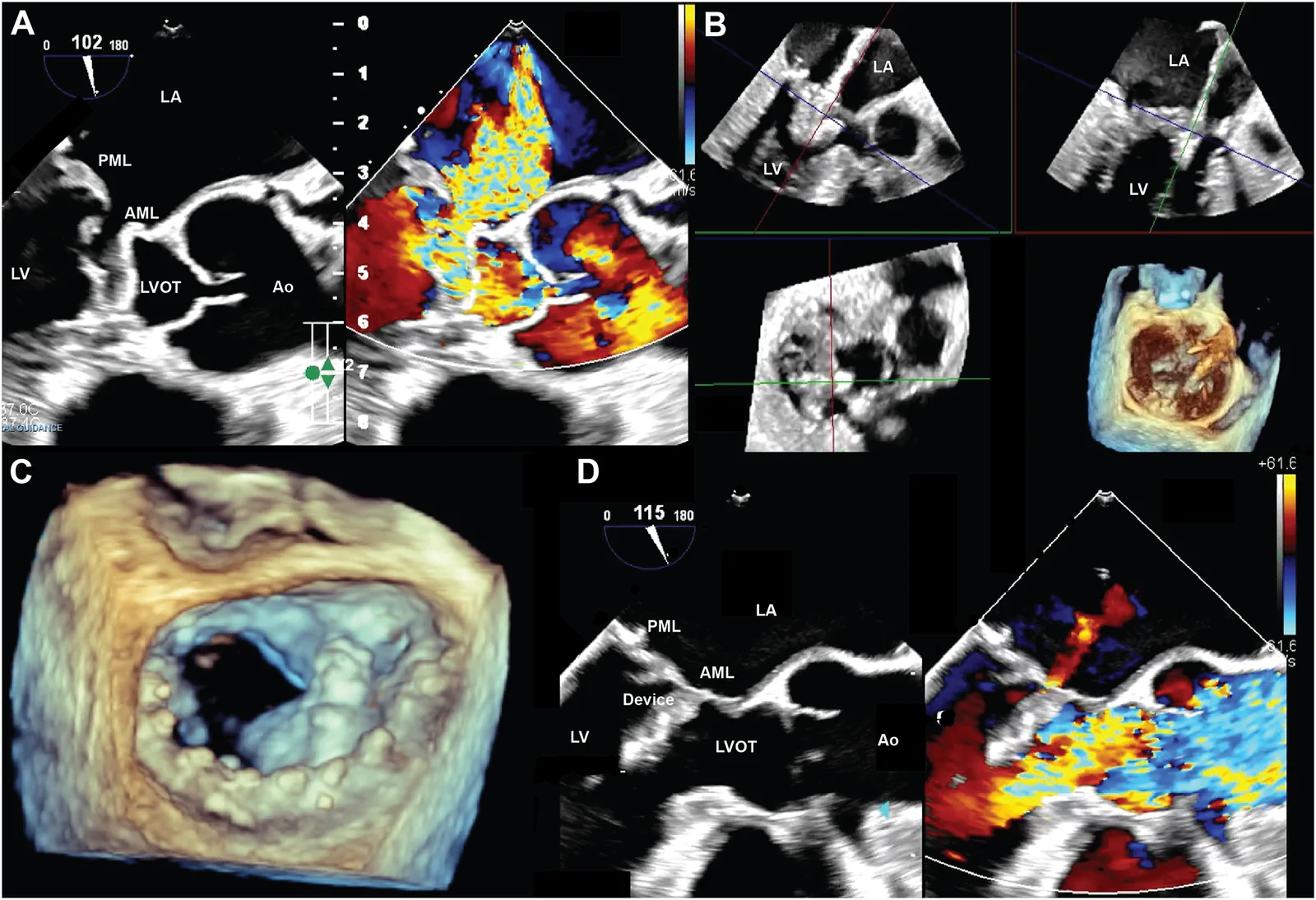
**SAM-mediated LVOT obstruction following TAVR treated with mitral TEER (case 4).** (**A**) Midesophageal long-axis TEE view demonstrating SAM of the mitral leaflets toward the interventricular septum, resulting in severe MR and dynamic LVOT obstruction. (**B**) Procedural guidance using multiplanar reconstruction showing mitral TEER device positioned between A3 and P3 coaptation plane. (**C**) 3D TEE en-face view of the mitral valve after PASCAL Ace deployment in diastole. (**D**) Imaging during isoproterenol provocation demonstrating resolution of SAM, trivial MR, and less turbulent LVOT flow following PASCAL Ace device implantation. Ao, aorta; AML, anterior mitral leaflet; LA, left atrium; LV, left ventricle; LVOT, left ventricular outflow tract; PML, posterior mitral leaflet.

**Figure 7:**
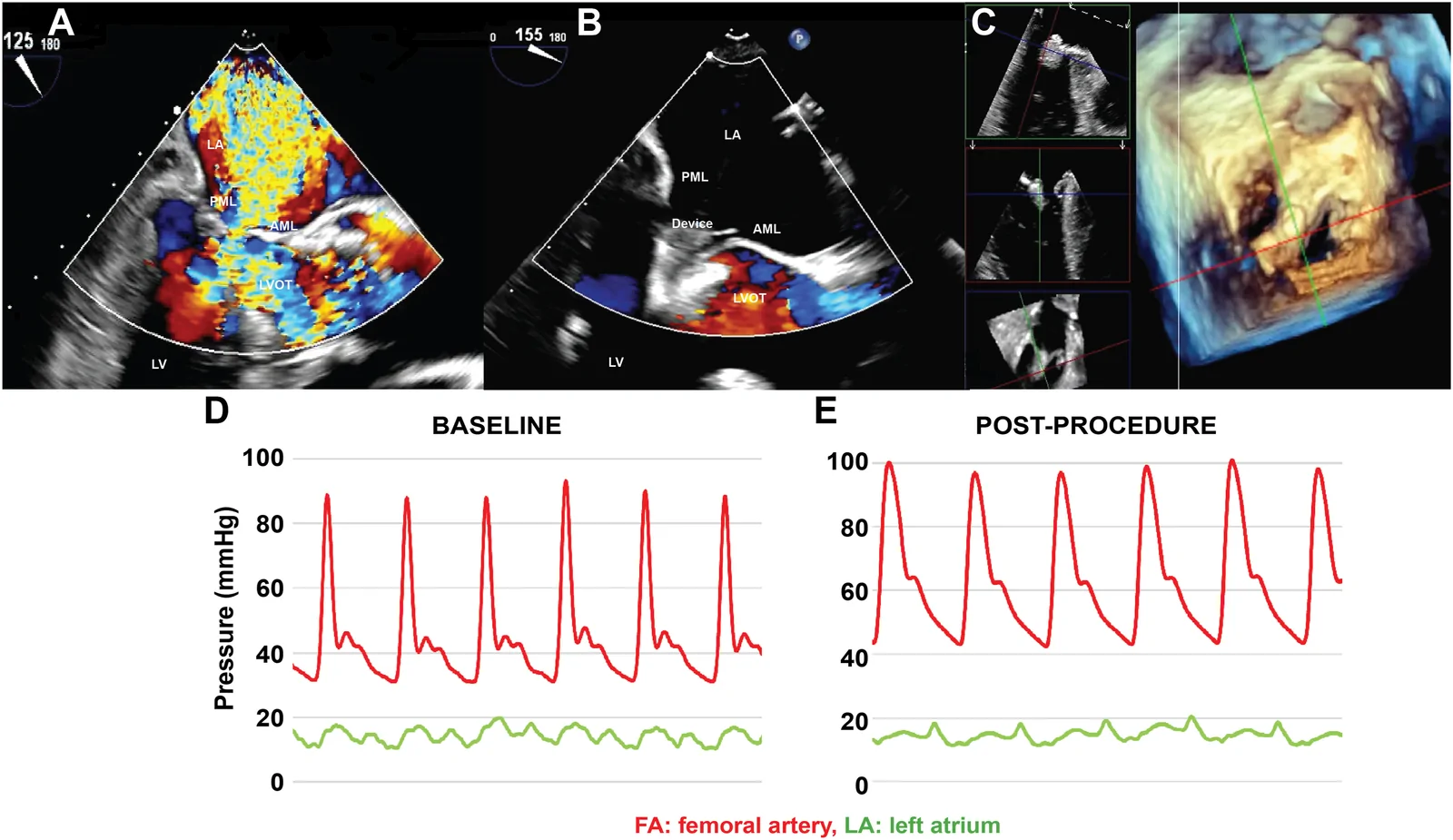
**SAM-mediated LVOT obstruction following TAVR treated with mitral TEER (case 5).** (**A**) Midesophageal long-axis TEE view demonstrating SAM-mediated severe, eccentric, posteriorly directed MR and LVOT obstruction with nitroglycerin provocation. (**B**) No residual MR and reduced turbulence of LVOT flow following PASCAL Ace device implantation (A2-P2). (**C**) Multiplanar reconstruction and procedural guidance. (**D**) Baseline invasive blood pressure recordings demonstrating dynamic LVOT physiology. (**E**) Improved systemic arterial pressure waveform and stable left atrial pressure following M-TEER and relief of SAM-mediated LVOT obstruction. AML, anterior mitral leaflet; FA, femoral artery; LA, left atrium; LV, left ventricle; LVOT, left ventricular outflow tract; PML, posterior mitral leaflet.

## Case 1

A 71-year-old man with HCM, prior alcohol septal ablation, and an extended septal myectomy presented with exertional dyspnea despite maximal tolerated beta blockade. Transthoracic echocardiography (TTE) showed SAM of an elongated anterior mitral leaflet with only mild dynamic obstruction (LVOT maximum instantaneous Doppler gradient 30 mm Hg with the Valsalva maneuver) and mild resting MR, discordant from the degree of his symptoms. Transesophageal echocardiography (TEE) with dobutamine confirmed severe dynamic LVOT obstruction (LVOT maximum instantaneous Doppler gradient > 150 mm Hg) and severe posteriorly directed MR. After heart team discussion, the patient opted to proceed with M-TEER. Isoproterenol provocation prior to intervention confirmed severe LVOT obstruction from SAM and severe MR (Figure 1A-D). A single PASCAL Ace device was positioned between the mid A2 and lateral P2 scallops (avoiding an incomplete cleft in the posterior leaflet) with resolution of SAM, mild residual MR, and no significant LVOT gradient (Figure 2A-E; Supplemental Video 1). The mean diastolic mitral valve gradient (MVG) was 5 mm Hg at a heart rate of 90 beats/min. At 1-year follow-up, the patient reported persistent improvement in symptoms (NYHA class I-II) with complete resolution of dynamic LVOT obstruction with mild residual MR.

## Case 2

An 82-year-old woman with HCM developed symptomatic LVOT obstruction and significant resting MR (SAM with P2 prolapse and a P1-P2 cleft) following transcatheter aortic valve replacement (TAVR) using a 23-mm Sapien 3 Ultra valve (Figure 3A-B). She had undergone tw2o alcohol septal ablations at 4 and 2 months prior to TAVR, with a decrease in maximum instantaneous LVOT gradient from 237 mm Hg to 5 mm Hg with Valsalva. A dual-chamber permanent pacemaker was implanted for complete heart block following the second septal ablation. However, on TTE 2.5 months post-TAVR, resting LVOT maximum instantaneous Doppler gradient was 38 mm Hg, rising to 52 mm Hg with Valsalva, with recurrent SAM-mediated MR and symptoms. After heart team discussion, M-TEER was undertaken for symptom relief. Invasive hemodynamics at rest and with isoproterenol provocation prior to M-TEER demonstrated worsening dynamic LVOT obstruction and severe MR (Figure 4A-B). She underwent M-TEER with a single PASCAL Ace device positioned between the A2-P2 scallops; her MR was reduced to trivial, and mean diastolic MVG was 4 mm Hg (Figure 3C-D; Supplemental Video 2). Postprocedural hemodynamics showed improved LVOT obstruction (Figure 4C). At 1 year, she remained well (NYHA class II) with stable TTE findings.

## Discussion

Patients in this series presented with SAM-mediated MR and dynamic LVOT obstruction arising from heterogeneous anatomic substrates. Across all 5 cases, restricted anterior leaflet excursion using off-label M-TEER effectively reduced SAM, alleviated dynamic LVOT obstruction, and improved symptoms without producing clinically significant mitral stenosis. All 5 patients achieved Mitral Valve Academic Research Consortium–defined technical success, ≤2+ residual MR, a reduction in LVOT gradient, and symptomatic improvement to NYHA class I to II at a median follow-up of 12 months.

To our knowledge, this is the first report of the PASCAL system (Edwards Lifesciences) for the treatment of SAM and dynamic LVOT obstruction. Precise anterior leaflet capture, secure leaflet retention, and device elongation that reduces chordal entanglement are particularly helpful given the small coaptation target and the need to avoid iatrogenic mitral stenosis. The outcomes of our series are consistent with a recent report of 35 HCM patients treated with the MitraClip system (Abbott).^7^ In our series, 60% (3/5) of patients had undergone prior septal reduction therapy, whereas only 9% (3/35) of patients in the previous registry had received prior septal ablation.^7^ Collectively, these findings suggest that in selected patients, M-TEER can obviate the need for septal reduction therapies. However, a nonsignificant trend toward increasing LVOT gradients over time in the registry, and the link between higher follow-up gradients and worse NYHA class, warrants caution and larger studies.^7^ Further research should clarify whether prior septal reduction influences long-term LVOT gradients and the optimal end point of M-TEER in HCM.

Sudden afterload reduction following TAVR may precipitate acute LVOT obstruction. Percutaneous options for refractory cases include alcohol septal ablation, right ventricular pacing, and M-TEER.^8^ In our series, 2 patients (cases 2 and 5) developed significant post-TAVR LVOT obstruction despite prior alcohol septal ablation, and both were successfully treated with M-TEER. Further studies are needed to evaluate the feasibility of a combined TAVR and M-TEER strategy in selected anatomies, which may reduce the need for prior septal reduction.

## Limitations

This small retrospective series has inherent limitations: patients were highly selected, and invasive hemodynamic assessment was not uniform. Follow-up ranged from 1 to 12 months, limiting conclusions regarding long-term durability. The benefits of cardiac myosin inhibitors in patients with SAM-mediated LVOT obstruction remain understudied.

## Conclusions

M-TEER using the PASCAL Ace device is a promising treatment strategy in selected patients with SAM and dynamic LVOT obstruction. Early outcomes were favorable across a range of LVOT phenotypes, but further studies are needed to better define anatomic criteria and durability.

## CRediT authorship contribution statement

**Mark J. Zorman:** Writing – review & editing, Writing – original draft, Visualization, Software, Resources, Project administration, Methodology, Investigation, Funding acquisition, Formal analysis, Data curation, Conceptualization. **Smitha Narayana Gowda:** Writing – review & editing, Formal analysis, Data curation. **Ratnasari Padang:** Writing – review & editing. **Jeremy J. Thaden:** Writing – review & editing. **Mayra Guerrero:** Writing – review & editing. **Mackram F. Eleid:** Writing – review & editing, Supervision, Conceptualization.

## Declaration of competing interest

Mayra Guerrero received research funds from Edwards Lifesciences. All other authors have no conflict of interest to report.

## Supplementary material

Supplementary Videos 1-5 Procedural transesophageal imaging from Cases 1–5.
